# Well-Being: Its Relationship with Work-to-Family Conflict and Burnout among Males and Females

**DOI:** 10.3390/ijerph16132291

**Published:** 2019-06-28

**Authors:** Shu-Ling Huang, Ren-Hau Li, Shu-Yi Fang, Feng-Cheng Tang

**Affiliations:** 1 Department of Psychology, Chung Shan Medical University, Taichung 402, Taiwan; 2 Room of Clinical Psychology, Chung Shan Medical University Hospital, Taichung 402, Taiwan; 3 Department of Leisure Services Management, Chaoyang University of Technology, Taichung 413, Taiwan; 4 Department of Occupational Medicine, Changhua Christian Hospital, Changhua 500, Taiwan; 5 School of Medicine, Chung Shan Medical University, Taichung 402, Taiwan; 6 School of Medicine, Kaohsiung Medical University, Kaohsiung 807, Taiwan

**Keywords:** work-to-family conflict, burnout, mental health, workplace health promotion, gender

## Abstract

The present study aims to apply gender-specific analyses to examine how work-to-family conflict (WFC) and burnout are related to well-being among the workers in Taiwan. A cross-sectional research design was adopted. A questionnaire was distributed to obtain information pertaining to demographic characteristics, WFC, burnout, and well-being. In total, 4259 full-time workers in the manufacturing industry were recruited. Gender-specific statistical analyses were used. The results showed that no significant gender difference occurred on WFC; however, females had higher scores on burnout compared to males. In the correlation analyses, WFC as well as burnout were negatively associated with well-being in both genders. In the regression analyses when demographic factors were controlled, burnout explained larger variances of well-being in both genders compared with WFC. WFC made a smaller contribution to the models predicting well-being in males in contrast to females. Moreover, the significant association between WFC and well-being for males disappeared when burnout was taken into account. The conclusion reached was that to improve workers’ well-being, organizations should develop relevant policies to decrease the extent of burnout for different genders. The policies that the organization adopted should consider females’ needs beyond work-related burden. Moreover, merely decreasing the extent of WFC is insufficient to enhance males’ well-being.

## 1. Introduction

During the past decades, the concept of subjective well-being has received particular attention with many social scientists trying to determine what factors contribute to people experiencing their lives in positive ways. Definitions of subjective well-being have been classified into different categories including external criteria such as virtue, standards of life satisfaction, and pleasant and unpleasant emotional experiences [1]. Diener et al. offered a broad concept (including life satisfaction, and positive and negative affects) and emphasized that the interaction of psychological factors with life circumstances in producing feelings of well-being warrants further study [2]. In the field of occupational health, many countries have promoted workers’ health in the workplace since the Declaration of Alma-Ata [3,4]. One of the main aims of the promotion of health in the workplace is to improve the level of well-being among workers rather than only to reduce illness. As a result of this interest in workers’ well-being, a large body of literature has been produced on the nexus of work and family life [5]. In addition, burnout is another issue mentioned frequently in research when workers’ well-being is focused [6,7,8]. The mechanism by which burnout is theorized to affect workers’ well-being has been generally described as resulting from a depletion of the burned-out individual’s personal resources that leads to a decline in one’s affective state [9].

According to previous studies, work–family conflict (the conflict between work life and family life) is a type of inter-role conflict in which role pressures in the work and family domains are mutually incompatible [10,11]. Work–family conflict was initially treated as a one-dimensional construct. Later, bi-directional constructs, i.e., work-to-family conflict (WFC) and family-to-work conflict, were proposed and considered to be distinct but related constructs [12]. Another approach suggested that conflict should not be considered to be the only factor and that the positive effects of combining work and family roles should be considered; i.e., work-to-family enrichment and family-to-work enrichment [13,14]. Among these constructs, widespread dysfunctionality and the costly social effects of WFC on work-related, non-work-related, and stress-related outcomes were found to be manifested [15]. The present study therefore focuses exclusively on the topic of WFC. Cultural traditions have unavoidably influenced individuals’ perception of the conflict between work and family [16]. More studies conducted in different countries are needed to complete the understanding of WFC. WFC has been associated with diminished satisfaction and lower levels of psychological well-being [17,18]. WFC as a predictor of employees’ well-being was supported in a longitudinal study in which social desirability bias was controlled [19]. To improve workers’ well-being, the issue of WFC should be considered as a part of workplace health promotion programs. 

Since Freudenberger’s and Maslach’s pioneering work, burnout has been widely addressed in the literature and there may well be a connection between WFC and burnout [20,21]. Certainly, more research is needed to gain a better understanding of burnout among Taiwanese workers. Burnout may be defined as a state of physical, emotional, and mental fatigue and exhaustion caused by excessive and prolonged stress and has been found to be associated with physical illness and concomitant absenteeism, poor general and mental health, lack of vitality, lower job satisfaction, and the intention to resign [22,23,24,25]. It has been shown that the negative impact of burnout on both employees and organizations is considerable, but in today’s highly competitive business world it is becoming increasingly difficult for employees to avoid burnout. Several studies in burnout among Taiwanese workers have been conducted, but in these the research sample was mainly limited to specific industries such as education and the healthcare service [26]. The levels of burnout among workers in the manufacturing industry have been rather overlooked.

In Taiwan, the number of females in the paid workforce has steadily increased during the past two decades, and had reached 50.6% when the present study was carried out [27,28]. However, a previous study reported that more females were employed in jobs where there was a high demand and/or with low decision-making latitude compared to males [29,30]. A study measuring diurnal variation in stress hormones found that stress levels in female managers were as high after work as they were during work, whereas among male managers, stress levels rapidly decreased after work [31]. Taiwanese females’ domestic caring responsibilities can be very demanding, which also adversely affects employment [32]. It seems that males and females may have very different experiences of both work life and family life. Therefore, a gender-specific analysis was adopted in the present study.

At the time this research was conducted, more than a third of paid workers were employed in the manufacturing industry [33]; therefore programs promoting their well-being is recommended. The present study aims to use gender-specific analyses to examine the association of WFC and burnout with the well-being among workers in the manufacturing industry in Taiwan.

## 2. Materials and Methods 

### 2.1. The Participants and Recruitment

The present study was conducted from 2013 to 2014 by the Center for Workplace Health Promotion, using a cross-sectional research method with convenience sampling. Workers 20 years of age or older were recruited from six manufacturing companies in Central Taiwan to participate in the study. The companies were chosen on the basis of their good relationship with the Center for Workplace Health Promotion. This allowed the study to proceed without difficulties. Their principal activities included manufacturing of electronic components, food, pumps, motor vehicle parts, and transport equipment. Before the investigation was carried out, an announcement was made to all the workers in those companies, requesting their voluntary participation. Potential participants were invited to complete the self-administrated questionnaire anonymously and to contribute their opinions on worksite health promotion. A consent form was distributed with the survey questionnaire. A total of 4957 full-time Taiwanese employees agreed to voluntarily take part in the study.

The study was conducted according to the Declaration of Helsinki and was approved by the Institutional Review Board of the Changhua Christian Hospital in Taiwan with a waiver of informed consent (CCHIRB No: 130508).

### 2.2. Measures

The survey questionnaire comprised four parts: personal information, WFC, burnout, and well-being. Personal information included gender, age, marital status, education, occupation, and economic status. Occupation was divided into two categories: white-collar worker (including management, professional, technician, office worker, and service worker) and blue-collar worker (including crafts worker and machine operator) [34]. Using a five-point Likert response format ranging from 1 “very poor” to 5 “excellent”; economic status was measured by one question: “How would you rate your economic status at the present time?” 

#### 2.2.1. Work-to-Family Conflict

The level of work-to-family conflict (WFC) was measured using five questions which were adopted from the work–home interference scale [35]. This scale assessed both time-based and strain-based conflict and exhibited good internal reliability with Cronbach’s alpha 0.820. The items were: “How often do you take your work home?”; “How often do you think about your work when you are at home?”; “How often does your work schedule conflict with your family/social life?”; “How often does your work leave you too tired to do things with your family/friends?” and “How often do you put your work before your home life?” This questionnaire used a four-point scale ranging from 1 (never) to 4 (always). Higher scores indicated higher levels of WFC. This questionnaire was chosen because the items were not limited to married individuals. The Cronbach’s alpha was 0.692 in the present study.

#### 2.2.2. Burnout

The level of burnout was measured by the Copenhagen Burnout Inventory (CBI) [22]. The CBI is a 19-item questionnaire measuring three burnout sub-dimensions. The personal burnout subscale has six questions measuring the degree of physical and psychological fatigue and exhaustion experienced by a person, regardless of his/her participation in the workforce. The work-related burnout subscale has seven questions measuring the degree of physical and psychological fatigue related to work. The client-related burnout subscale has six questions measuring the degree of physical and psychological fatigue specifically related to working with clients. Responses were made on a 5-point scale ranging from 0 (never) to 4 (always). The scale labels were then re-coded into 0 (never), 25, 50, 75, and 100 (always). Higher scores indicated more burnout. All three subscales were found to have very high internal reliability and satisfactory validity in studies [22,36]. According to the theoretical rationale, the three subscales can be used independently in accordance with the populations being studied [22]. For the purposes of the present study, only the subscales of personal and work-related burnout were adopted to form a mean as general burnout. In this study, Cronbach’s alpha was 0.947 for general burnout.

#### 2.2.3. Well-being

The short version of the Chinese Happiness Inventory, a validated instrument, was adopted to measure the level of well-being [37]. This scale was adapted from the Oxford Happiness Inventory with some modifications to fit the Taiwanese culture [38]. It had 10 items regarding positive affect, lack of negative affect, and life satisfaction. Each item comprised four statements. The participants were requested to check one to represent their feelings of well-being. This scale was on a four-point Likert scale. A higher score indicated a higher level of well-being. For this study, the alpha reliability was 0.921.

### 2.3. Statistical Analysis

The demographic characteristics of the participants, along with their data pertaining to WFC, burnout, and well-being were summarized using descriptive statistics. The chi-square test or t-test was used to compare the differences in demographic characteristics and other surveyed variables by gender. Pearson’s correlation was used to analyze the relationships between the continuous variables for male and female workers separately. Gender-specific multiple linear regression in hierarchical ways was used to investigate the individual and collaborative relations of WFC and burnout to well-being. Among the controlled factors, not married and white-collar were coded as 0; married and blue-collar were coded as 1. All statistical procedures were performed using SPSS statistics 20 (SPSS Inc., Chicago, IL, USA); a p-value less than 0.05 was considered statistically significant.

## 3. Results

In total, 4957 questionnaires were distributed. After excluding the data with more than 20% of question items uncompleted to ensure the credibility of the results, 4,259 were successfully collected. Table 1 lists the demographic characteristics, WFC, burnout, and well-being of the participants by gender. Of the participants, 82.1% were males and 17.9% were females. The majority of the participants were aged 50–59 (37.4%) or 30–39 (32.9%), married (76.0%), and white-collar workers (74.9%). About two-thirds of the participants perceived their economic status as ordinary (65.8%). The comparisons of age, educational level, occupation, economic status, burnout, and well-being by gender showed significant differences. Female employees showed higher scores on burnout and well-being compared to male employees.

The correlations of research variables are listed in Table 2. WFC was significantly positive with burnout. WFC and burnout show significantly negative relations with well-being. 

Multiple linear regressions predicting well-being are summarized in Table 3. For males, in model A1, the model for demographic factors predicting well-being was significant (F = 155.07, *p* < 0.001; R^2^ = 0.184). In model A2, with demographic factors controlled, WFC showed a significantly negative association with well-being (*β* = −0.098, *p* < 0.001); the R^2^ was 0.193 (F = 137.27, *p* < 0.001). In model A3, with demographic factors controlled, burnout showed a negative association with well-being (*β* = −0.474, *p* < 0.001); the R^2^ increased to 0.373 (F = 342.16, *p* < 0.001). In model A4, burnout was negatively related to well-being (*β* = −0.477), whereas WFC was not significantly associated with well-being (F = 293.32, *p* < 0.001; R^2^ = 0.374). For females, in model B1, demographic factors predicting well-being was significant (F = 25.80, *p* < 0.001; R^2^ = 0.147). In model B2, with demographic factors controlled, WFC showed a significantly negative association with well-being (*β* = −0.239, *p* < 0.001; F = 31.20, *p* < 0.001; R^2^ = 0.200). In Model B3, with demographic factors controlled, burnout showed a significantly negative association with well-being (*β* = −0.501, *p* < 0.001; F = 70.29, *p* < 0.001; R^2^ = 0.360). In model B4, both WFC and burnout were negatively related to well-being (*β* = −0.081 and *β* = −0.471 separately); the R^2^ was 0.366 (F = 61.59, *p* < 0.001).

## 4. Discussion

The main purpose of this study was to evaluate the relations of WFC and burnout to well-being after adjusting with the covariates for males and for females, separately. The results showed that in the multiple linear regression analyses, when WFC (19.3% for males and 20.0% for females) was compared with burnout (37.3% for males and 36.0% for females), burnout explained larger variances of well-being with bigger standardized regression coefficients as well. With regard to gender difference, WFC made a smaller contribution to the models predicting well-being in males (∆R^2^ = 0.009) in contrast with females (∆R^2^ = 0.053). Moreover, the significant effect of WFC on the well-being for males virtually disappeared when burnout was taken into account. There were no obvious differences between males and females in terms of the effects of burnout.

Since the 1980s, an understanding of the importance of WFC has been steadily increasing because employees want to achieve a balance between their work and family lives [15]. WFC has been found to be significantly associated with psychological distress [15]. Previous studies have also reported the adverse effects of WFC on job satisfaction, turnover rate, general health, severe musculoskeletal pain, and severe sleep disorders [15,39]. However, it is worth noting that the relationships between WFC and well-being were not prominent in the present study, particularly for male workers. Merely decreasing the levels of WFC might be insufficient to enhance male workers’ well-being.

The levels of personal burnout (mean = 37.7) and work-related burnout (mean = 37.5) in the present study were above the average scores found in the PUMA study (35.9 for personal burnout and 33.0 for work-related burnout). The PUMA study, which considered 15 jobs, was a Danish longitudinal study titled “Project on Burnout, Motivation and Job Satisfaction” in which the CBI was developed [22]. Another national survey conducted in Taiwan in 2004 with a large sample found significant gender differences in both personal and work-related burnout [40]. In that survey, male employees scored 34.7 for personal burnout and 28.9 for work-related burnout; female employees scored 36.8 for personal burnout and 29.8 for work-related burnout. In the present study, we found a score of 37.3 for both personal and work-related burnout in males, and in females 39.6 for personal burnout and 38.2 for work-related burnout. These were all above the figures of the previous survey. In particular, the scores for work-related burnout in both genders have showed a remarkable increase during the past decade. In the present study, burnout was clearly related to well-being in both genders. In addition, burnout was found to have significantly adverse effects on professional identity, work engagement and mental health [41,42,43]. Strategies to mitigate burnout among Taiwan’s employees should be adopted as a matter of urgency. In addition, it is worth noting that personal burnout in females was significantly higher than that in males, though WFC and work-related burnout did not show gender differences. Possibly, female workers had more non-work related burden compared to male workers. Therefore, the strategies that companies develop to decrease burnout should consider female workers’ non-work related needs.

Despite differing research results, most scholars agree that females show higher levels of burnout than male workers both in Taiwan and in other countries [40,41,44,45,46,47]. Our results show a similar phenomenon. However, while this study shows that females have higher levels of burnout than males, it also shows that females have a greater sense of well-being than males. Previous studies indicate that females tend to be employed in positions with higher levels of stress than males and also usually undertake the majority of household duties [29,30,32,48]. Nevertheless, a demanding level of domestic work in itself is not necessarily harmful for people’s mental health [49,50]. People’s resilience may well offer an alternative perspective and would be worth exploring. In general, gender dissimilarities in people’s health might be mediated by several variables, such as age, ethnicity, education, working conditions, domestic situations, or social role [50,51]. From the view of Taiwanese culture, three kinds of obedience (to father before marriage, to husband after marriage, and to son after the death of husband) and four types of virtues (morality, proper speech, modest manner, and diligent handiwork) are considered as Taiwanese women’s behavior standards since the Sung Dynasty of ancient China. The issue regarding how and to what extent the specific cultural conditioning leads to accepted gender roles in the modern life is worth further clarifying. In addition, no significant gender difference in WFC was found in the present study. This is consistent with another study in Taiwan [52]. Notably, we found that WFC showed stronger associations with well-being in females than in males. A previous study suggested that WFC was more strongly associated with exhaustion in females than in males [53]. Females seem to be more affected by work stress than males [48,54]. It may be that female workers are more sensitive to both negative affect and positive affect compared to male workers.

To the best of our knowledge, this is the first paper to empirically examine the simultaneous association of WFC and burnout to well-being in Taiwan. The results of the study have shed some light on the importance of implementing strategies in the workplace to counteract WFC and burnout for promoting workers’ mental health. However, there were some limitations to the study. First, the cross-sectional study design did not allow the determination of the developmental process and causal relationship among the research variables. Some longitudinal studies have nevertheless found strain-based work–family interference to be the precursor to poor health but reversed causation and a bi-directional relationship cannot be ruled out [55,56]. Second, a certain number of eligible participants chose not to participate in the present study. However, nonresponsive bias could not be assessed due to data being obtained anonymously. In addition, the data in this study were obtained a few years ago and the research companies were selected previously. Therefore, applying the research results should be done with caution. Third, the slightly low reliability of WFC might restrict the inference of the relationships between WFC and well-being. Developing an appropriate instrument to measure WFC for Taiwanese workers is recommended for future research.

## 5. Conclusions

The present study applies an empirical, gender-specific analysis to examine how WFC and burnout are related to the well-being among the workers in Taiwan. The results showed that no significant gender differences occurred on WFC; however, females had higher scores on burnout compared to males. In the multiple linear regression models, burnout explained larger variances of well-being than WFC in both genders. WFC made a smaller contribution to the models predicting well-being in males compared to females. In addition, the significant relationship between WFC and well-being for males disappeared when burnout was taken into account. Merely decreasing the extent of WFC is insufficient to enhance workers’ well-being, particularly for male workers. To enhance workers’ well-being, decreasing the extent of burnout could be a more reasonable approach. In addition, the levels of work-related burnout in men and in women in Taiwan have increased considerably over the last ten years. This issue should not be overlooked. Moreover, females seemed to have more personal burden compared to males. Therefore, the strategies that companies develop to decrease burnout should consider female workers’ needs beyond work-related burden.

## Figures and Tables

**Table 1:** Comparing demographic characteristics and research variables by gender.

	Total (n = 4259)	Male (n = 3495)	Female (n = 764)	
Variables	n (%) ^a^	n (%) ^a^	n (%) ^a^	χ^2^
Age (year)				220.531 ***
20–29	321 (7.5)	270 (7.7)	51 (6.7)	
30–39	1401 (32.9)	1254 (35.9)	147 (19.2)	
40–49	785 (18.4)	507 (14.5)	278 (36.4)	
50–59	1592 (37.4)	1325 (37.9)	267 (34.9)	
60 and over	160 (3.8)	139 (4.0)	21 (2.7)	
Marital status				0.187
not married	1018 (23.9)	840 (24.0)	178 (23.3)	
married	3241 (76.1)	2655 (76.0)	586 (76.7)	
Education				46.600 ***
junior high	56 (1.3)	29 (0.8)	27 (3.5)	
senior high	922 (21.7)	759 (21.8)	163 (21.4)	
university	2674 (62.9)	2178 (62.5)	496 (65.1)	
postgraduate	597 (14.1)	521 (14.9)	76 (10.0)	
Occupation				12.850 ***
white-collar	3237 (76.0)	2618 (74.9)	619 (81.0)	
blue-collar	1022 (24.0)	877 (25.1)	145 (19.0)	
Economic status				46.921 ***
very poor	92 (2.2)	81 (2.3)	11 (1.5)	
poor	405 (9.6)	365 (10.6)	40 (5.3)	
ordinary	2775 (65.8)	2291 (66.2)	484 (63.9)	
good	771 (18.3)	578 (16.7)	193 (25.5)	
excellent	174 (4.1)	144 (4.2)	30 (4.0)	
**Variables (possible range)**	**Mean (SD)**	**Mean (SD)**	**Mean (SD)**	***t***
WFC ^b^ (5–20)	9.2 (2.3)	9.2 (2.3)	9.1 (2.2)	1.39
Burnout (0–100)	37.6 (17.3)	37.3 (17.4)	38.9 (16.5)	-2.30 *
Personal burnout (0–100)	37.7 (18.5)	37.3 (18.6)	39.6 (17.9)	-3.17 **
Work-related burnout (0–100)	37.5 (17.4)	37.3 (17.6)	38.2 (16.7)	-1.3
Well-being (0–30)	13.4 (5.3)	13.2 (5.4)	13.9 (5.0)	-3.30 **

^a^ Calculated according to a percentage of the valid count. ^b^ WFC denotes work-to-family conflict. * *p* < 0.05; ** *p* < 0.01; *** *p* < 0.001 calculated using *t* or χ^2^ test.

**Table 2:** The correlations of research variables in male and female workers.

	Male		Female	
Variables	WFC ^a^	Burnout	WFC ^a^	Burnout
WFC ^a^	--		--	
Burnout	0.246 **	--	0.330 **	--
Well-being	−0.113 **	−0.564 **	−0.256 **	−0.565 **

^a^ WFC denotes work-to-family conflict. ** *p* < 0.01.

**Table 3:** Summary of multiple linear regressions predicting the well-being for male and female workers.

	Male				Female			
	Model A1	Model A2	Model A3	Model A4	Model B1	Model B2	Model B3	Model B4
	*β*	*β*	*β*	*β*	*β*	*β*	*β*	*β*
Age	0.184 ***	0.177 ***	0.054 **	0.054 **	0.186 ***	0.185 ***	0.066 *	0.073 *
Marital Status ^a^	0.066 ***	0.075 ***	0.093 ***	0.092 ***	0.065	0.075 *	0.078 *	0.080 **
Education	0.001	0.013	0.003	0.002	−0.011	0.031	−0.028	−0.013
Occupation ^a^	−0.058 ***	−0.063 ***	−0.050 ***	−0.050 **	0.017	−0.006	0.027	0.018
Economic Status	0.287 ***	0.279 ***	0.178 ***	0.178 ***	0.271 ***	0.235 ***	0.153 ***	0.147 ***
WFC ^b^		−0.098 ***		0.010		−0.239 ***		−0.081 *
Burnout			−0.474 ***	−0.477 ***			−0.501 ***	−0.471 ***
Total R^2^	0.184	0.193	0.373	0.374	0.147	0.200	0.360	0.366
∆R^2^		0.009	0.189	0.190		0.053	0.213	0.219
F	155.07 ***	137.27 ***	342.16 ***	293.32 ***	25.80 ***	31.20 ***	70.29 ***	61.59 ***

*β* denotes standardized regression coefficient. ^a^ Not married and white-collar were coded as 0; married and blue-collar were coded as 1. ^b^ WFC denotes work-to-family conflict. * *p* < 0.05; ** *p* < 0.01; *** *p* < 0.001.
